# Biological Roles of Fibroblasts in Periodontal Diseases

**DOI:** 10.3390/cells11213345

**Published:** 2022-10-24

**Authors:** Koji Naruishi

**Affiliations:** Department of Periodontology and Endodontology, Institute of Biomedical Sciences, Tokushima University Graduate School, Tokushima 770-8504, Japan; naruishi@tokushima-u.ac.jp; Tel.: +81-88-631-3111; Fax: +81-88-633-7009

**Keywords:** gingival fibroblasts, periodontal ligament fibroblasts, periodontitis, drug-induced gingival overgrowth

## Abstract

Periodontal diseases include periodontitis and gingival overgrowth. Periodontitis is a bacterial infectious disease, and its pathological cascade is regulated by many inflammatory cytokines secreted by immune or tissue cells, such as interleukin-6. In contrast, gingival overgrowth develops as a side effect of specific drugs, such as immunosuppressants, anticonvulsants, and calcium channel blockers. Human gingival fibroblasts (HGFs) are the most abundant cells in gingival connective tissue, and human periodontal ligament fibroblasts (HPLFs) are located between the teeth and alveolar bone. HGFs and HPLFs are both crucial for the remodeling and homeostasis of periodontal tissue, and their roles in the pathogenesis of periodontal diseases have been examined for 25 years. Various responses by HGFs or HPLFs contribute to the progression of periodontal diseases. This review summarizes the biological effects of HGFs and HPLFs on the pathogenesis of periodontal diseases.

## 1. Introduction

Human gingival fibroblasts (HGFs) are the most abundant cells in gingival connective tissue [1]. Human periodontal ligament fibroblasts (HPLFs) are located between the teeth and the alveolar bone, namely, the “periodontal ligament”, and contribute to the stable embedding of teeth [2]. These cells are typical fibroblasts in periodontal tissues, and they maintain the homeostasis of connective tissue through the secretion and degradation of components of the extracellular matrix, such as collagen. Proteases secreted by fibroblasts, including matrix metalloproteases (MMPs), are involved in the degradation of the extracellular matrix and bone collagen matrix [3]. Although various MMPs, including MMP-1, -3, -8, -9, and -13, are involved in the remodeling of periodontal tissue [3], MMP-1 and MMP-3 may have important roles because collagen types I & III are dominant in periodontal connective tissues [3,4]. Collagen proteins degraded into pieces or fragments are transported in the lysosomes of fibroblasts via phagocytosis and are ultimately digested to amino acids by the lysosome enzymes cathepsins B and L [5].

Periodontitis is a chronic inflammatory disease that is triggered by periodontal bacteria, such as *Porphyromonas gingivalis* (*Pg*) [6]. Its pathogenesis is regulated by many inflammatory cytokines, including interleukins (IL)-1 and -6 [7]. IL-1 and IL-6 are pro-inflammatory cytokines that play central roles in the early phase of periodontitis [7,8]. HGFs and HPLFs have been shown to facilitate the inflammatory cascade in periodontitis [8]. In contrast, gingival overgrowth is a side effect of some drugs, such as cyclosporin, phenytoin (PHT), and calcium channel blockers [9]. Although the excessive destruction of gingival connective tissue due to collagen degradation occurs in periodontal lesions [10], the accumulation of large amounts of collagen occurs in gingival overgrowth lesions [11]. Therefore, periodontitis and gingival overgrowth may both be characterized by an imbalance in collagen metabolism. In general, it has been considered that the pathogenesis of drug-induced gingival overgrowth is very complicated and involves various host/immune cells [12]. Gingival overgrowth may be clinically characterized by fibrosis with some degree of inflammation because of poor plaque control induced by the formation of false deep periodontal pockets caused by overgrown gingiva, and accordingly, the author has chosen to focus more narrowly on drug-induced gingival growth. HGF responses that are mediated by drugs, but not inflammation, are important for clarifying the pathogenesis of onset of gingival overgrowth.

The roles of HGFs and HPLFs in the pathogenesis of periodontal diseases, namely, periodontitis and drug-induced gingival overgrowth, are discussed in this review.

## 2. Chronic Periodontitis

Chronic periodontitis is an inflammatory disease that destroys the tissue structures supporting teeth, leading to tooth loss [13]. The onset of periodontitis is involved in the infection of periodontal bacteria in periodontal pockets [6]. A recent study reported that periodontitis, a persistent low-grade infection by Gram-negative bacteria, increased the incidence of systemic diseases, such as diabetes mellitus (DM) and atherosclerosis, because of the invasion of bacterial pathogens into the circulation [14]. Low-grade bacteremia-induced inflammatory markers, including C-reactive protein and IL-6, were elevated in the serum of patients with periodontitis, resulting in the development of DM and atherosclerosis [15,16]. Therefore, periodontitis is attracting increasing attention.

### 2.1. Effects of Lipopolysaccharide (LPS) Derived from Pg

Although more than 700 bacterial species are present in the oral cavity, the Gram-negative bacterial pathogen *Pg* has been strongly implicated in the onset of periodontitis [17]. *Pg* possesses a number of virulence factors, such as LPS, gingipains, and fimbriae [18,19]. LPS is a cell wall component of Gram-negative bacteria that initiates various inflammation cascades in periodontal lesions [20]. A previous study demonstrated that TLR2 and TLR4 were both expressed in HGFs [21]. Although the specific receptor for *Pg*-LPS in HGFs has not yet been identified, HGFs may be a target cell of *Pg*-LPS.

*Pg*-LPS activates several intracellular proteins, including mitogen-activated protein kinase (MAPK), IL-1 receptor-associated kinase (IRAK), nuclear factor-κB (NF-κB), and activating protein-1 (AP-1) in HGFs and HPLFs [22,23,24]. Many investigators have reported that *Pg*-LPS induces the production of pro-inflammatory cytokines, i.e., IL-1, IL-6, IL-8, and tumor necrosis factor alpha (TNF-α), via the NF-κB pathway in HGFs [25,26,27]. HPLFs also perform a similar function to that of HGFs [28,29]. Furthermore, HPLFs share some of the properties of both osteoblasts and cementoblasts [30]. Kato showed that *Pg*-LPS inhibited ALP activity, COL1A1 and osteocalcin production, and mineralization in HPLFs, and induced the production of IL-1β, IL-6, and IL-8 [31]. HPLFs stimulated with *Pg*-LPS have been suggested to suppress the regeneration of periodontal tissue and promote the progression of periodontitis.

### 2.2. Effects of Pro-Inflammatory Cytokines

#### 2.2.1. IL-1β

Increased levels of IL-1β in gingival crevicular fluids (GCFs) were found to correlate with the severity of periodontitis [32]. IL-1β is a multifunctional cytokine associated with immune and inflammatory responses, and is also a critical factor for bone and connective tissue destruction because it enhances the collagenolytic activity of fibroblasts [33].

Two types of receptors bind IL-1β: type I receptors (IL-1RI) and type II receptors (IL-lRII) [34]. IL-1RI transduces IL-1β signals into the cytoplasmic domain, whereas IL-1RII is a “decoy” target of IL-1β. A previous study reports that IL-1β signals via IL-1RI were significantly decreased in IL-1RII-overexpressing HGFs [35]. After IL-1β-IL-1RI binding, several intracellular signaling molecules, such as MAPK, NF-κB, and AP-1, were activated via the phosphorylation of IRAK in HGFs [36].

IL-1β has been shown to induce the expression of TNF-α, IL-6, and IL-8 via the MAPK and NF-κB pathways in HGFs and HPLFs [37,38,39]. IL-1β may play a central role in regulating the inflammatory cascade surrounding fibroblasts in the early stage of periodontitis. A previous study reports that IL-1β strongly enhanced the collagenase activity of HGFs, whereas that of HPLFs was rarely increased above that of the basal control [40]. On the other hand, the collagenase activity of HPLFs stimulated with IL-1β was suggested to be more sensitive than that of HGFs in the presence of tissue inhibitor of MMPs (TIMPs) [41]. There is currently no established theory to explain the superiority of collagen degradation by HGFs and HPLFs.

#### 2.2.2. IL-6

Increased levels of IL-6 and IL-1β have been detected in periodontal lesions [42]. IL-6 plays a crucial role in inflamed periodontal tissue, osteoclast differentiation, and continuous bone resorption [43]; therefore, its role may partially overlap with that of IL-1β.

IL-6 binds to the cell-surface IL-6 receptor gp80, and these molecules build a complex with the IL-6 signal transducer gp130, which, in turn, activates the intracellular signaling pathway [44]. HGFs cannot be a target of IL-6 because they do not constitutively express sufficient levels of gp80 to bind appreciable amounts of IL-6 [1]. Therefore, the soluble form of the IL-6 receptor (sIL-6R), which only consists of the extracellular domain of gp80, is essential for IL-6 to bind to HGFs [1,45]. The IL-6/sIL-6R complex induces intracellular signaling via the phosphorylation of gp130 in HGFs, and this event activates at least two distinct signaling cascades: Janus kinase/signal transducer and activator of transcription signaling, and MAPK signaling [45].

Yamaguchi reported that IL-6/sIL-6R enhanced the production of cathepsins B and L in HGFs via the caveolin-1-mediated JNK-AP-1 pathway [46]. Since cathepsins B and L also degrade gingival collagen fibers [33], both MMPs and cathepsins released from HGFs may promote the progression of periodontitis. IL-1β has been shown to significantly up-regulate the expression of gp130 and IL-6 in HGFs [39]. Therefore, IL-1β enhances the IL-6 responsiveness of HGFs by autocrine loops. IL-6/sIL-6R synergistically induced the expression of MMP-1, -3, -13, and -14, IL-1ra, IL-33, MCP-1, bFGF, and VEGF in HGFs pretreated with IL-1β [39]. The synergistic responses of HGFs by IL-1β and IL-6/sIL-6R may promote periodontal inflammation by inducing the expression of gp130, resulting in the progression of periodontitis.

Macrophages that infiltrate in inflamed periodontal tissues produce sIL-6R. Previous studies have demonstrated that IL-6, calprotectin, and high glucose significantly up-regulates the expression of sIL-6R in THP-1 macrophages [39,47]. Further studies are warranted to explain the importance of HGF-macrophage crosstalk in the pathophysiology of periodontitis mediated by IL-6.

#### 2.2.3. Other Inflammation-Related Molecules

S100 calcium-binding protein A8 (S100A8) and S100A9 are mainly released from neutrophils under inflammatory conditions [48]. S100A8 and S100A9 may form homo- or hetero-complexes, the latter of which is known as calprotectin. Increased levels of calprotectin have been observed in the GCFs of patients with periodontitis [49]. Nishikawa demonstrated that calprotectin markedly increased IL-6 and MCP-1 production in HGFs via TLR4 signaling [50]. As described above, since calprotectin induces the secretion of sIL-6R in macrophages, calprotectin-mediated HGF-macrophage crosstalk may enhance the IL-6 responsiveness of HGFs, leading to the progression of periodontitis.

TNF-α induced type 2 soluble form of TNF receptor (sTNFR2), but not sTNFR1 in HGFs treated with IL-1β, resulting in the inhibition of TNF-α binding to HGFs [51]. Since sTNFR2 is an antagonist of TNF-α, the effects of TNF-α on HGFs may be synergistically suppressed by the influence of IL-1β. Although TNF-α and IL-1β are both proinflammatory cytokines, the synergism of TNF-α and IL-1β surrounding HGFs may inhibit inflammation responses in the acute phase.

Transforming growth factor-β (TGF-β) produced in the early phases of wound healing up-regulated the expression of MMP-3 and -13 and procollagen in HGFs via the p38 MAPK pathway [52,53]. MMP-13 is considered to be involved in the rapid turnover of connective tissue during gingival wound repair [53]. Furthermore, Fujii showed that TGF-β1 promoted cell proliferative activity, the expression of α-smooth muscle actin (α-SMA), and type I collagen in HPLFs [54]. The controlled degradation of the extracellular matrix is required for wound repair in inflamed periodontal tissues. TGF-β may play an important role in wound healing or regeneration in the periodontal ligament by targeting HPLFs.

### 2.3. Significant Roles of Fibroblasts in Severe Periodontitis in Diabetic Patients

DM is a systemic disease with several complications, such as retinopathy, nephropathy, neuropathy, and periodontitis [55]. Poor glycemic control has been clinically associated with the severity of periodontitis. Inversely, increases in the persistent invasion of low-grade inflammatory cytokines or periodontal pathogens into the bloodstream may be a key factor aggravating DM in patients with severe periodontitis [56]. In this section, the possible mechanisms underlying the aggravation of periodontitis in patients with DM are discussed with a focus on the effects of diabetic factors, such as high glucose and advanced glycation end-products (AGEs), on the responses of HGFs.

#### 2.3.1. High Glucose

Omori previously reported that high glucose up-regulated the expression of gp130 in HGFs [57]. IL-6/sIL-6R also up-regulated the expression of vascular endothelial growth factor (VEGF) in HGFs cultured under high-glucose conditions via the ERK-C/EBP pathway [57]. VEGF is a strong inducer of angiogenesis. Since VEGF levels in gingival tissues were found to be higher in diabetic patients with periodontitis compared to non-diabetic patients [58], VEGF released from HGFs may be an important mediator of severe periodontitis in diabetic patients. High glucose also significantly increased the IL-1β and IL-6/sIL-6R-induced production of MMP-1 in HGFs via the ERK or NF-κB pathway [47]. High glucose impaired both the proliferation and migration of HGFs [59]. In DM patients with periodontitis, delayed wound healing by dysfunctions in HGFs may occur, as well as increases in the production of VEGF and MMP-1 in HGFs.

HPLFs have been shown to express higher levels of the cellular fibronectin receptor under high glucose conditions [60]. Furthermore, high glucose suppressed the cathepsin activity of HPLFs [61]. This may explain the accumulation of the extracellular matrix because cathepsins are the main proteases responsible for collagen metabolism. Possible increases in the cellular fibronectin receptor and the accumulation of collagen by high glucose may enhance the adhesiveness of HPLFs to matrix proteins in non-inflamed gingival tissues, which may, in turn, reduce cell migration into inflamed lesions.

#### 2.3.2. AGEs

AGEs are stable metabolic proteins that are non-enzymatically glycated and oxidized, and which are synthesized not only under diabetic conditions, but also by inflammation or aging [62]. AGEs are strong inducers of diabetic complications, including diabetic nephropathy and neuropathy [62,63]. An increase in AGEs was detected in the gingival tissues of DM patients with periodontitis [64]. These pathological findings indicate a role for AGEs in the progression of periodontitis. A specific receptor of AGEs in various cells, including HGFs, is the receptor for AGE (RAGE) [63]. Previous studies showed that AGEs induced the expression of MMP-1, IL-6, and ICAM-1 in HGFs via the RAGE-NF-κB pathway [65,66]. Since AGEs induced apoptosis and autophagy in HPLFs through the production of reactive oxygen species (ROS) [67], the ROS-induced autophagy of HPLFs may be a new therapeutic target in diabetic patients with severe periodontitis.

## 3. Drug-Induced Gingival Overgrowth

Gingival overgrowth is an unwanted side effect of systemic medication [68]. At least three types of drugs have been reported to enlarge the gingiva, i.e., the immunosuppressive drug cyclosporine A (CsA), the anti-epileptic drug PHT, and the calcium antagonist nifedipine (Nif) [69]. Gingival overgrowth is associated with esthetic issues and poor oral hygiene, which, in turn, increase susceptibility to periodontitis. Gingival enlargement is generally attributed to an imbalance between the synthesis and degradation of the extracellular matrix, mainly collagen [3]. The excessive accumulation of collagen in gingival connective tissue has been attributed to increases in collagen production and to decreases in collagen degradation and subsequent intracellular digestion by HGFs. Furthermore, it is important to note that the pathogenesis may be complex because of poor plaque control induced by the formation of false deep periodontal pockets caused by overgrown gingiva. Therefore, drug-induced gingival overgrowth is clinically characterized by fibrosis with some degree of inflammation. HGF responses that are mediated by drugs, but not inflammation, are important for clarifying the pathogenesis of gingival overgrowth. However, the molecular mechanisms underlying drug-induced fibrosis have not yet been elucidated in detail.

The following two routes have been proposed for collagen degradation: 1. an extracellular pathway by the activation of MMPs secreted by fibroblasts; 2. an intracellular pathway by the lysosomal cathepsins of fibroblasts, after the phagocytosis of digested collagens. Maita previously showed that the secretion of MMP-1 was significantly inhibited in HGFs cultured with CsA, PHT, and Nif [70]. I-cell disease (mucolipidosis II) is a rare congenital lysosomal disease characterized by impaired lysosomal enzymes, including cathepsins [71]. Patients with I-cell disease manifest several characteristic features, including gingival overgrowth [72]. Since the lysosomal cysteine proteinases cathepsins are responsible for the digestion of extracellular proteins, the development of gingival overgrowth may be attributed to impaired cathepsin activity and subsequent matrix accumulation. Mice deficient in the cathepsin-L gene were found to have an enlarged gingiva, similar to gingival overgrowth [73]. Yamada showed that the activity of cathepsin L, but not cathepsin B, was significantly inhibited in HGFs cultured with CsA, PHT, and Nif [74]. Based on impaired MMP-1 and cathepsin L activities in HGFs cultured with CsA, PHT, and Nif, these drugs may have common target molecules in HGFs, resulting in gingival overgrowth. Although the underlying mechanisms remain unclear, many investigators have attempted to understand these attractive research subjects by targeting HGFs for more than 25 years. Further studies with the aim of preventing the unwanted common side effects of CsA, PHT, and Nif are warranted.

### 3.1. The Immunosuppressant CsA

CsA is an immunosuppressive drug that is frequently used in organ transplants to prevent graft rejection [75]. It binds to the intracellular receptors, immunophilins [76], and their complex blocks the activation of calcium/calmodulin-dependent phosphatase, known as calcineurin [77].

Arora reported that CsA markedly suppressed collagen degradation by inhibiting the intracellular phagocytic activity of HGFs [78]. Furthermore, CsA significantly inhibited the production of MMP-1, MMP-3, and cathepsin-L in HGFs [74,79]. The inhibition of collagen degradation by HGFs treated with CsA may be attributed, at least in part, to reductions in the production of MMPs and cathepsins. In addition, Omori showed that cAMP-response element binding protein (CREB) regulated the CsA-mediated down-regulation of cathepsin B and L synthesis in HGFs [80]. CREB may be an attractive target of CsA-induced gingival overgrowth in HGFs, because calcineurin is a phosphatase that regulates the phosphorylation of CREB [81]. Furthermore, Chung found that CsA increased type I collagen production in HGFs by up-regulating the TGF-β pathway [82]. This finding indicates the potential of CsA as a direct inducer of collagen accumulation, as observed in patients with gingival overgrowth, by targeting HGFs.

### 3.2. The Anticonvulsant PHT

PHT is an antiepileptic drug that is used widely in the treatment of epilepsy [83], and affects voltage-gated sodium, calcium, and potassium ion channels. Yamada reported a positive correlation between the daily drug dose and PHT levels in the sera of patients with gingival overgrowth; however, no significant differences were observed in the infection levels of periodontal pathogens between the gingival overgrowth and non-gingival overgrowth groups [84]. The metabolic activity of PHT may play an important role in the development of PHT-induced gingival overgrowth.

PHT significantly inhibited the production of MMP-1, MMP-3, and cathepsin-L in HGFs [74]. Kato also showed that PHT strongly prevented collagen phagocytosis by HGFs via the suppression of α2β1-integrin expression [85]. A series of studies revealed that PHT induced the accumulation of collagen by inhibiting enzymatic degradation and phagocytosis in HGFs, leading to gingival overgrowth. Furthermore, PHT decreased the gene and protein expression of MMP-1, but increased the gene expression of TIMP-1 in HPLFs; however, it currently remains unclear whether HPLF responses are involved in the development of gingival overgrowth [86]. PHT was also shown to induce the migration of and osteogenic differentiation in HPLFs, leading to accelerated periodontal healing. PHT may promote wound healing mediated by HPLFs. Periostin is a TGF-β-induced matricellular protein that is expressed in collagen-rich tissues, such as periodontal connective tissues, and regulates the synthesis of the extracellular matrix during periodontal healing [87]. Kim reported that PHT up-regulated the expression of periostin in HGFs via the phosphorylation of Smad3 [88]. Although periodontal healing induced by PHT may depend on the collaboration of HGFs and HPLFs, PHT-induced matrix production including periostin by HGFs is more important in the pathogenesis of gingival overgrowth.

### 3.3. The Calcium Channel Blocker Nif

Calcium channel blockers, such as Nif, have frequently been used as effective drugs in patients with hypertension [89]. Corresponding to the worldwide increase in patients with hypertension, Nif is now more frequently used in clinical settings [69].

Nif has been shown to increase the synthesis of collagen in HGFs [90,91]. It also up-regulated the expression of TGF-β in HGFs [92]. Kim reported that Nif up-regulated the expression of periostin in HGFs in a TGF-β-dependent manner [93]. On the other hand, it significantly inhibited MMP-1 production and cathepsin-L activity in HGFs [70,73], leading to impairments in the extra- and intracellular digestion of collagen. These findings indicate that Nif directly induces the accumulation of collagen in periodontal connective tissues by targeting HGFs.

A clinical study previously demonstrated the higher expression of androgen receptors in Nif-induced overgrown gingiva than in healthy gingiva [94]. Although the role of androgen receptors in Nif-induced gingival overgrowth remains unclear, Nif was shown for the first time to up-regulate the expression of procollagen α1(I) in HGFs via the activation of androgen receptors. Since NF-κB plays an important role in the expression of androgen receptors in HGFs [95], inflammation-related molecules, such as IL-1 and IL-6/sIL-6R, may indirectly be strong inducers of Nif-mediated gingival overgrowth.

## 4. Conclusions

**Figure 1:**
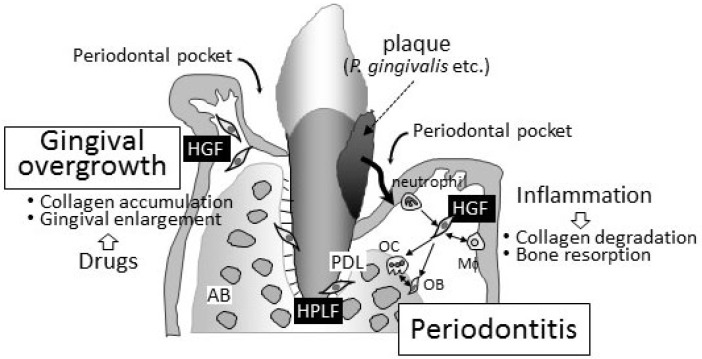
Schematic representation of periodontitis and gingival overgrowth. Periodontitis is an infectious/inflammatory disease caused by periodontal bacteria. The main clinical symptom is collagen degradation and bone resorption. Both HGFs and HPLFs play important roles in the progression of periodontitis. Neutrophils and macrophages also infiltrate inflamed periodontal tissues, and the importance of cell–cell interactions surrounding HGFs has been proposed in the pathophysiology of periodontitis. On the other hand, gingival overgrowth is characterized by an accumulation of collagen in periodontal connective tissues caused by three drugs, i.e., immunosuppressants, anticonvulsants, and calcium channel blockers. No bone resorption can be found in the lesion of gingival overgrowth. HGFs that are affected by these drugs, but not HPLFs, play an important role in the progression of gingival overgrowth. Periodontal pockets grow gradually deeper corresponding to the progression of periodontitis. Deepening periodontal pocket is the most important finding of periodontitis. Periodontal pocket seen in gingival overgrowth is secondary, and periodontitis is frequently found in patients with gingival overgrowth clinically. HGF, human gingival fibroblast; HPLF, human periodontal ligament fibroblasts; AB, alveolar bone; PDL, periodontal ligament; OB, osteoblasts; OC, osteoclasts.

The pathogeneses of periodontitis and drug-induced gingival overgrowth in gingival connective tissue are completely contrasting (Figure 1). The degradation/destruction of the extracellular matrix in connective tissue has been detected in periodontal lesions. On the other hand, the accumulation of the extracellular matrix has been reported in gingival overgrowth. Although drug-induced gingival overgrowth may occur with “inflammation” in periodontal connective tissues in clinical settings, periodontitis is a secondary issue induced by the infection of periodontal bacteria into the deep periodontal pockets caused by the enlargement of gingival tissues. Fibroblasts in both the gingiva and periodontal ligament, namely, HGFs and HPLFs, play a key role in regulating the pathogenesis of both diseases. These cells are attractive targets for clarifying the pathophysiology of these unwanted diseases.

## Data Availability

Not applicable.
